# Anticancer Potential of Cannabidiol in Renal Cell Carcinoma: Serum Modulation and Preliminary Mechanistic Insights

**DOI:** 10.3390/jcm15020792

**Published:** 2026-01-19

**Authors:** Débora Sousa, Filipa Amaro, Ana Margarida Araújo, Márcia Carvalho

**Affiliations:** 1 Instituto de Investigação, Inovação e Desenvolvimento Fernando Pessoa (FP-I3ID), Fernando Pessoa University, Fernando Pessoa Teaching and Culture Foundation, Praça de 9 de Abril 349, 4249-004 Porto, Portugal; debora.beatriz.sousa@gmail.com; 2 Associate Laboratory i4HB—Institute for Health and Bioeconomy, University of Porto, 4050-313 Porto, Portugal; up201608003@up.pt; 3 UCIBIO—Applied Molecular Biosciences Unit, Laboratory of Toxicology, Department of Biological Sciences, Faculty of Pharmacy, University of Porto, 4050-313 Porto, Portugal; 4 Laboratório Associado para a Química Verde/Rede de Química e Tecnologia (LAQV/REQUIMTE), Laboratory of Bromatology and Hydrology, Department of Chemical Sciences, Faculty of Pharmacy, University of Porto, 4050-313 Porto, Portugal; 5 RISE-Health, Faculty of Health Sciences, Fernando Pessoa University, Fernando Pessoa Teaching and Culture Foundation, Rua Carlos da Maia 296, 4200-150 Porto, Portugal

**Keywords:** cannabidiol, renal cancer, antitumoral activity, redox modulation, serum

## Abstract

**Background**: Cannabidiol (CBD), the major non-psychotropic cannabinoid derived from *Cannabis sativa* L., has demonstrated broad anticancer activity across multiple tumor types; however, its effects in renal cell carcinoma (RCC) remain largely undefined. Given the ongoing need for novel therapeutic strategies in RCC, this study provides preliminary mechanistic insights into the cytotoxic, antiproliferative, and redox-modulating properties of CBD in RCC cells and evaluates the influence of serum conditions on its activity. **Methods**: Human RCC cell lines (Caki-1 and 769-P) and non-tumoral proximal tubular epithelial cells (HK-2) were treated with CBD (1–100 µM) for up to 48 h under serum-free and serum-supplemented (5%) conditions. Cytotoxic and antiproliferative effects were assessed using the MTT assay, and intracellular reactive oxygen/nitrogen species (ROS/RNS) levels were quantified using the H_2_DCFDA fluorescence assay. **Results**: CBD significantly decreased RCC cell viability and proliferation in a concentration-dependent manner and induced time-dependent ROS/RNS accumulation. Comparable sensitivity was observed in non-tumoral HK-2 renal epithelial cells, indicating limited tumor selectivity under the tested in vitro conditions. Notably, these effects were markedly attenuated in the presence of serum, consistent with CBD’s high serum–protein binding and reduced free bioavailability. **Conclusions**: CBD induces cytotoxic, antiproliferative, and redox-modulating effects in RCC cells in vitro; however, these responses are strongly attenuated by serum, lack tumor selectivity, and require concentrations exceeding clinically achievable plasma levels. Together, these findings delineate major translational limitations for the therapeutic use of CBD in RCC.

## 1. Introduction

Cannabidiol (CBD), a major phytocannabinoid isolated from *Cannabis sativa* L., is recognized for its broad spectrum of pharmacological actions, including anti-inflammatory, antioxidant, and significant anticancer effects [1,2,3]. Unlike Δ^9^-tetrahydrocannabinol (THC), CBD is devoid of psychotropic (“high”) effects, which substantially enhances its suitability for therapeutic development [4]. Emerging preclinical evidence has consistently demonstrated CBD’s antitumoral activity across various malignancies, such as glioma/glioblastoma [5,6], breast [7,8], prostate [9,10], lung [11,12], and colorectal [5,10] cancers, often through interference with multiple cellular and molecular mechanisms implicated in tumorigenesis and cancer progression.

The breadth of these antitumoral effects reflects CBD’s distinctive pharmacological profile, which is characterized by a multitargeted and pleiotropic mode of action [4]. In contrast to THC, CBD displays negligible affinity for the classical G-protein-coupled cannabinoid receptors CB_1_ and CB_2_. Rather than acting through canonical cannabinoid signaling, many of its biological effects are mediated through indirect modulation of the endocannabinoid system and direct interactions with a variety of non-cannabinoid receptors and signaling pathways [3,4,13,14]. Among the most relevant non-cannabinoid targets are transient receptor potential (TRP) channels, particularly TRPV1 and TRPV2, which are activated by CBD. Activation of TRPV2 has been shown to induce apoptosis in lung cancer cells and to enhance chemotherapy-induced apoptosis in breast cancer models [7,8]. CBD also antagonizes the G-protein-coupled receptor 55 (GPR55), a receptor implicated in tumor growth and invasiveness, and its inhibition has been associated with reduced proliferation in pancreatic cancer cells [15]. In addition, CBD interacts with nuclear receptors such as peroxisome proliferator-activated receptor gamma (PPARγ), contributing to transcriptional regulation of genes involved in cell cycle control and apoptosis [11]. Modulation of serotonin (5-HT) receptors has also been proposed to contribute to CBD-mediated regulation of cellular stress responses [3,16]. Despite these mechanistic insights across multiple tumor types, the cellular effects of CBD in renal cancer models remain incompletely characterized.

Renal cell carcinoma (RCC), the most frequent renal epithelial malignancy, accounts for 2–3% of all human cancers [17,18]. Its increasing frequency and global distribution make it a public health priority. Despite recent therapeutic advances, including the use of targeted agents such as tyrosine kinase inhibitors (TKIs) and immune checkpoint inhibitors [19], these treatments rarely achieve complete tumor eradication and are frequently limited by resistance and recurrence [20]. Consequently, there is an urgent need to investigate novel therapeutic targets and strategies for RCC management. Notably, RCC is characterized by profound metabolic reprogramming, driven in part by dysregulation of the VHL/HIF axis, including altered mitochondrial metabolism, increased dependence on glutamine, and an enhanced antioxidant buffering capacity that supports survival under oxidative stress [21,22] and contributes to therapy resistance [23]. These metabolic adaptations suggest that agents capable of disrupting redox homeostasis and mitochondrial function, such as CBD, may be particularly relevant in this tumor context by exploiting intrinsic metabolic vulnerabilities.

To date, CBD has not been investigated in RCC, despite its well-established pro-oxidant and mitochondrial-disruptive actions in other cancer models, and may therefore represent a promising therapeutic candidate. Additionally, because CBD exhibits high serum–protein binding, which may restrict its cellular availability and therapeutic efficacy, it is essential to clarify how serum conditions influence its biological actions. Accordingly, the present study provides the first experimental evidence that RCC cells are responsive to CBD, offers preliminary mechanistic insights into its antitumoral activity, and establishes a foundation for the prospective development of CBD as a redox-oriented therapeutic strategy for RCC.

## 2. Materials and Methods

### 2.1. Chemicals

All reagents were of analytical grade and obtained from certified suppliers. Dulbecco’s Modified Eagle Medium (DMEM), fetal bovine serum (FBS), penicillin–streptomycin (10,000 U/mL and 10,000 μg/mL, respectively), and dimethyl sulfoxide (DMSO) were purchased from PAN-Biotech (Aidenbach, Germany). 2′,7′-Dichlorodihydrofluorescein diacetate (H_2_DCFDA) was obtained from Sigma-Aldrich (St. Louis, MO, USA). All other chemicals, including trypsin–EDTA, were purchased from Biowest (Nuaillé, France). Cannabidiol (CBD; 99% purity) was obtained from Meet Harmony Limited (London, UK). Stock solutions were prepared in DMSO and freshly diluted in culture medium prior to each experiment, maintaining a final DMSO concentration ≤0.05% (*v*/*v*) in all assays.

### 2.2. Cell Lines and Culture Conditions

Human renal carcinoma cell lines Caki-1 (ATCC^®^ HTB-46) and 769-P (ATCC^®^ CRL-1933), along with the non-tumoral proximal tubular epithelial cell line HK-2 (ATCC^®^ CRL-2190), were obtained from the American Type Culture Collection (ATCC, Manassas, VA, USA). Cells were routinely cultured in DMEM supplemented with 10% FBS and 1% penicillin–streptomycin. Although hypoxic conditions may more closely reflect the RCC tumor microenvironment, all experiments were conducted under standard normoxic conditions (21% O_2_, 5% CO_2_, 37 °C) to ensure experimental consistency across assays. Cells were used within the following passage ranges: HK-2, passages 21–36; Caki-1, passages 32–38 and 49–55; and 769-P, passages 13–23.

### 2.3. Cell Viability and Proliferation Assays

The effects of CBD on cell viability and proliferation were assessed using the MTT assay. For cytotoxicity (viability) measurements, cells were seeded in 96-well plates at a density of 1.2 × 10^4^ cells per well and allowed to adhere for 24 h, ensuring near-confluent conditions prior to treatment; no serum starvation was applied during the seeding or adhesion period. Cells were then exposed to CBD at final concentrations of 1, 1.5, 2, 3, 4, 5, 7.5, 10, 15, 20, 30, 40, 50, and 100 µM for 24 or 48 h under either serum-free (0% FBS) or serum-supplemented (5% FBS) conditions. The reduced-serum condition (5% FBS), commonly used in in vitro studies evaluating the anticancer effects of CBD [16], was selected to limit excessive growth factor signaling while maintaining cell viability.

The antiproliferative effects of CBD were evaluated using a long-term growth assay designed to monitor cell proliferation over an extended period (up to 72 h). To prevent premature confluence and allow accurate assessment of growth dynamics, cells were seeded at a lower initial density of 0.6 × 10^4^ cells per well. Following overnight attachment, cells were treated with CBD at IC_10_ and IC_25_ concentrations (derived from 24 h concentration–response curves) and maintained in serum-free or serum-supplemented (5% FBS) medium for 24, 48, and 72 h. Cell proliferation was quantified by measuring MTT reduction at baseline (0 h, prior to treatment) and at each subsequent time point.

At the end of each exposure period, the culture medium was replaced with an MTT solution (0.5 mg/mL), and cells were incubated at 37 °C for 1.5 h as previously described [24]. Formazan crystals were solubilized in DMSO, and absorbance was measured at 545 nm (reference at 630 nm) using a Varioskan LUX multimode microplate reader (Thermo Scientific, Waltham, MA, USA). Cell viability was expressed as a percentage of the untreated (negative) control, with cells treated with 1% Triton X-100 serving as the positive control.

All experiments were performed in at least three independent biological replicates for each time point.

### 2.4. Measurement of Intracellular ROS/RNS Levels

Intracellular reactive oxygen/nitrogen species (ROS/RNS) production was quantified using H_2_DCFDA fluorescence, as previously described [24]. RCC and HK-2 cells were seeded in 96-well plates under the same conditions used for the cell viability assay and allowed to adhere overnight. The culture medium was then removed, and cells were loaded with 100 µL of H_2_DCFDA solution (100 µM) per well. After incubation for 30 min at 37 °C in the dark, the probe solution was removed, and cells were treated with CBD at final concentrations of 5, 10, or 15 µM.

Intracellular ROS/RNS levels were assessed exclusively under serum-supplemented conditions (5% FBS), as serum deprivation alone was found to induce significant cellular stress. Fluorescence intensity (excitation 485 nm, emission 530 nm) was measured at 0.5, 1.5, 3, 5, 24, and 48 h using a Synergy HTX multimode plate reader (BioTek, Winooski, VT, USA). Data represent three independent experiments performed in triplicate and are expressed as fluorescence intensity normalized to untreated controls.

### 2.5. Statistical Analysis

All statistical analyses and nonlinear regression models were performed using GraphPad Prism version 10.6.1 (GraphPad Software, San Diego, CA, USA). Cell mortality curves were constructed as a function of the logarithm of the concentration (µM), based on data obtained from the MTT assay. The curve that best fit the experimental data was modeled according to the equation: Y = 100/(1 + 10^((LogIC50 − X) × HillSlope)^), where Y represents the normalized response (cell mortality, %), X corresponds to the logarithm of the CBD concentration (µM), LogIC_50_ represents the logarithm of the CBD concentration that induces 50% cell mortality, and HillSlope describes the slope of the curve. Comparisons between the 24 h and 48 h mortality curves were performed using the extra sum-of-squares F test. Data on antiproliferative activity and ROS/RNS production are presented as mean ± standard error of the mean (SEM). Data normality was assessed using the Shapiro–Wilk test. To evaluate the effects of treatment condition (control, IC_10_, IC_25_) and incubation time, a two-way ANOVA was conducted for each cell line, followed by Tukey’s post hoc test for multiple comparisons. Statistical significance was defined as a *p*-value < 0.05.

## 3. Results

### 3.1. CBD Elicits RCC Cell Death, with Stronger Efficacy Under Serum-Free Conditions

To capture biological heterogeneity within RCC, we investigated the effects of CBD in two complementary RCC models: Caki-1 cells, derived from a metastatic lesion, and 769-P cells, originating from a primary tumor [25,26]. These models differ in aggressiveness and metastatic potential, thereby providing a relevant framework to assess CBD-induced cytotoxicity across distinct RCC phenotypes.

**Figure 1:**
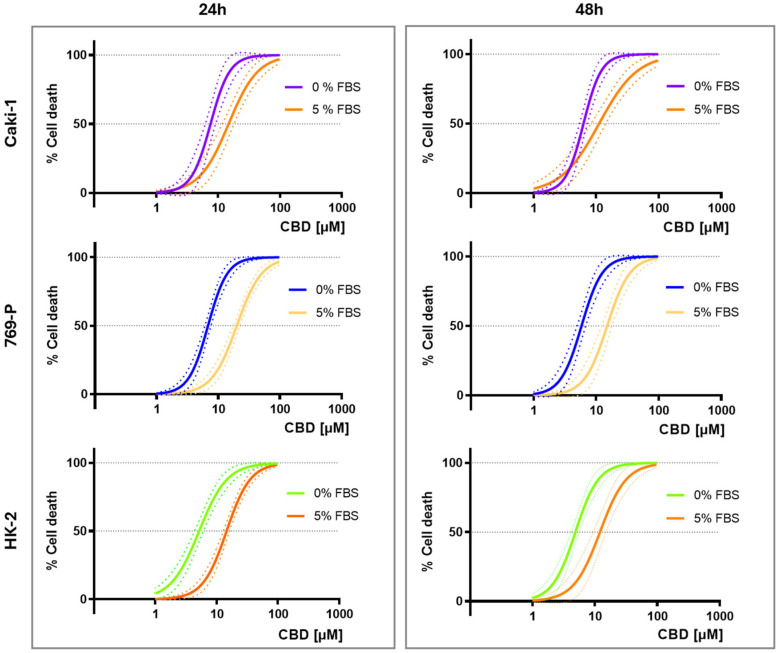
Nonlinear regression models for the cell death induced by CBD (1–100 µM) in Caki-1, 769-P, and HK-2 cells, under serum-free (0% FBS) and serum-supplemented (5% FBS) conditions, as evaluated by the MTT assay, after 24 h and 48 h of exposure. Dotted lines represent the 95% confidence band of each fit. Results were obtained from three independent experiments, performed in duplicate.

Within this experimental context, we first examined the effects of a broad concentration range (1–100 µM) of CBD on the viability of RCC and HK-2 cells up to 48 h under serum-free and serum-supplemented conditions. As shown in Figure 1, the concentration–response curves obtained by MTT reduction at 24 h and 48 h demonstrate a clear concentration-dependent increase in cell mortality across all cell lines. Consistent with the loss of cell viability, CBD-treated tumoral and non-tumoral cells exhibited marked morphological alterations, including membrane blebbing, cytoplasmic vacuolization, cell rounding, and detachment from the culture surface compared with control cells; these changes were increasingly evident at higher CBD concentrations.

Importantly, and consistent with the influence of extracellular factors on drug bioactivity, serum supplementation markedly attenuated this cytotoxic response, evidenced by a rightward shift of the mortality curves in 5% FBS compared with serum-free medium (*p* < 0.01, Figure 1). At 24 h, the IC_50_ values under 5% FBS were 14.5 µM for HK-2, 14.8 µM for Caki-1, and 20.1 µM for 769-P, whereas in serum-free medium, they decreased substantially to 5.2, 6.9, and 6.8 µM, respectively (*p* < 0.01, Table 1). Notably, the sensitivity of HK-2 cells to CBD was comparable to that of both RCC cell lines under the conditions tested (Figure 1, Table 1).

To determine whether prolonged exposure further influenced CBD-induced cytotoxicity, concentration–response profiles obtained at 24 h and 48 h were compared. No significant time-dependent effects were observed, except for 769-P cells maintained in serum-supplemented medium, in which a modest leftward shift of the curve was detected at 48 h (*p* = 0.0147) (Supplementary Figure S2). This observation is consistent with the nearly identical IC_50_ values obtained at both time points (Table 1), indicating that extended CBD exposure did not meaningfully enhance cytotoxicity and that most effects were already established within the first 24 h of treatment.

### 3.2. CBD Inhibits RCC Proliferation Without Tumor Selectivity

**Figure 2:**
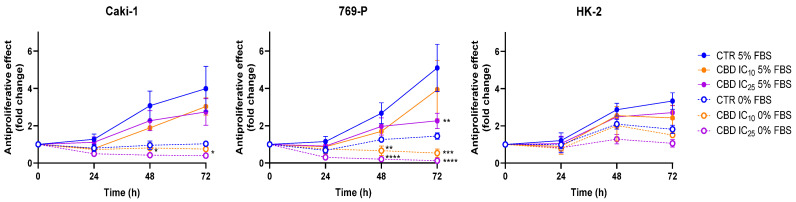
Proliferation kinetics of Caki-1, 769-P, and HK-2 cells exposed to CBD at 0, IC_10_, and IC_25_ concentrations for up to 72 h under serum-free (0% FBS) and serum-supplemented (5% FBS) conditions. In FBS-deprived medium, CBD IC_10_ values were 3.1, 2.8, and 1.5 µM, and IC_25_ values were 4.6, 4.2, and 2.8 µM for Caki-1, 769-P, and HK-2 cells, respectively. In FBS-supplemented medium, CBD IC_10_ values were 3.5, 7.2, and 4.7 µM, and IC_25_ values were 6.8, 12.0, and 7.9 µM for Caki-1, 769-P, and HK-2 cells, respectively. Data are presented as mean ± SEM from three independent experiments performed in triplicate. * *p* < 0.05, ** *p* < 0.01, *** *p* < 0.001, **** *p* < 0.0001 vs. respective control.

We next evaluated the impact of CBD on RCC cell proliferation. It is important to note that serum deprivation markedly reduces cell growth in all cell lines. As shown in Figure 2, both RCC cell lines exhibited complete serum dependence, with the absorbance values of control cells remaining practically unchanged over the 72 h period when cultured in serum-free medium. HK-2 cells retained some proliferative capacity under serum-free conditions, although their growth was still substantially lower than in FBS-supplemented cultures (Figure 2).

Regarding the effect of CBD, both RCC cell lines showed a clear, concentration-dependent trend toward reduced proliferation, reaching statistical significance in 769-P cells exposed to the higher concentration tested (IC_25_, *p* < 0.01). Although the effect of CBD on the non-tumoral HK-2 cells did not reach statistical significance, the magnitude of inhibition was comparable to that observed in the RCC cell lines, indicating a lack of tumor selectivity. Taken together, these findings suggest that CBD exerts broad cytotoxic and cytostatic effects across renal cell types, rather than preferentially targeting malignant cells.

### 3.3. CBD Induces Biphasic, Time-Dependent Effects on Intracellular ROS/RNS Levels

**Figure 3:**
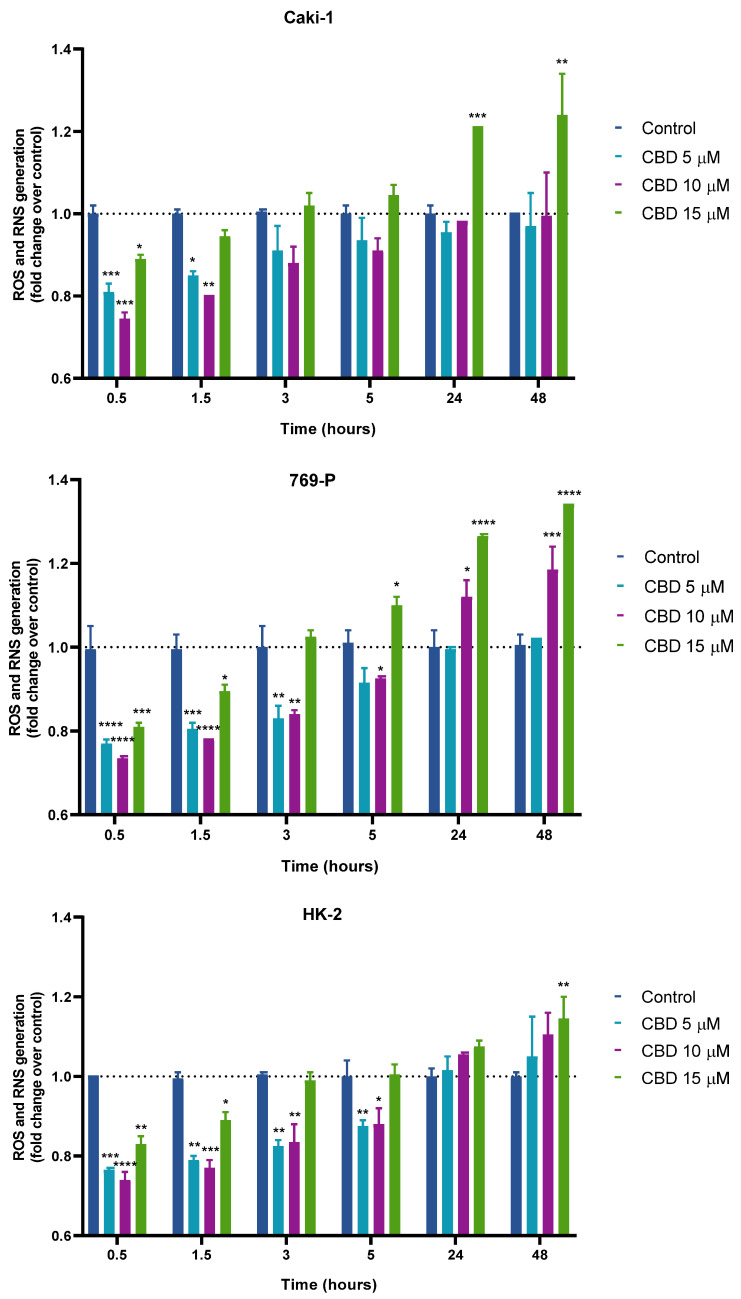
Effects of CBD (5–15 µM) on ROS/RNS production in Caki-1, 769-P, and HK-2 cells over 0.5–48 h under serum-supplemented conditions. The dashed line represents the control level set to 1-fold for ROS/RNS, serving as a reference for comparing treatment effects. Bars indicate mean ± SEM fold change relative to control from three independent experiments. * *p* < 0.05, ** *p* < 0.01, *** *p* < 0.001, **** *p* < 0.0001 vs. control.

We also investigated CBD’s ability to modulate intracellular redox homeostasis by assessing its effects on ROS/RNS production in renal cells. Analysis of ROS/RNS levels in Caki-1 and 769-P cells revealed a time-dependent, biphasic response to CBD (Figure 3). During the early incubation periods (0.5 and 1.5 h), ROS/RNS levels substantially decreased at all tested concentrations relative to control cells. In Caki-1 cells, this reduction was significant at 0.5 h for 5, 10 and 15 μM, and at 1.5 h for 5 and 10 μM (*p* < 0.05). Similarly, 769-P cells showed significant decreases at 0.5 and 1.5 h for all tested concentrations and at 3 h for 5 and 10 μM (*p* < 0.05). From 5 h onward, ROS/RNS levels began to rise, reaching statistical significance at 24 and 48 h in Caki-1 cells, particularly at 15 μM, and in 769-P cells at 5 h for 15 μM, as well as at 24 and 48 h for the highest concentrations tested (*p* < 0.05).

HK-2 cells exhibited a comparable, time-dependent pattern. During the early incubation periods (0.5–5 h), ROS production decreased relative to the control, with significant reductions at 5 μM and 10 μM across all time points (*p* < 0.05) and at 15 μM until 1.5 h (*p* < 0.01). From 5 h onward, ROS/RNS levels gradually increased, becoming particularly pronounced and statistically significant at 48 h for 15 μM CBD (*p* < 0.01). As observed in the RCC cell lines, CBD triggered an early antioxidant response in HK-2 cells that was later followed by a rise in ROS/RNS levels; however, the onset of this pro-oxidant shift occurred at a somewhat later point compared with the cancer cell lines (Figure 3).

## 4. Discussion

This study provides the first experimental evidence that CBD exerts direct cytotoxic and cytostatic effects in human RCC cell models (Caki-1 and 769-P) in a concentration-dependent manner. A key finding, consistent with previous cannabinoid research [5,7,9,27,28], was the marked attenuation of CBD-induced cell death when assays were conducted under serum-supplemented conditions compared to serum-free conditions. Although FBS is routinely included in in vitro cancer studies, serum reduction or withdrawal during drug exposure is frequently employed to minimize confounding effects of growth factors [10,29,30]. In the present study, the absence of serum substantially exacerbated CBD cytotoxicity, as evidenced by a pronounced decrease in IC_50_ values from 14.8 to 20.1 µM in serum-supplemented media to approximately 6.8 µM under serum-free conditions. This enhanced cytotoxicity likely reflects a combination of pharmacokinetic and cellular stress-related factors. CBD is known to bind extensively to plasma proteins (~95%) [31], thereby limiting the freely available fraction capable of cellular uptake and interaction with intracellular targets. At the same time, serum deprivation itself imposes metabolic and oxidative stress, alters redox homeostasis, and reshapes cellular bioenergetics, potentially sensitizing cells to additional insults [32,33,34,35]. Accordingly, part of the enhanced cytotoxicity observed under serum-free conditions may arise from synergistic stress responses rather than CBD-specific mechanisms alone, underscoring the need for cautious interpretation of data generated under non-physiological conditions.

Although the effective micromolar concentrations of CBD revealed in the present study fall within ranges commonly reported in other preclinical studies [16], the concentrations required to elicit cytotoxicity under serum-supplemented conditions (IC_50_ values up to ~20 µM) raise important translational considerations. Clinical pharmacokinetic studies have shown that oral administration of high CBD doses (1500 mg/day for 7 days or a single dose of 20 mg/kg) results in maximal plasma concentrations of only 3.2–5.2 µM [36,37], highlighting the limited systemic exposure achievable even at high dosing regimens. In addition, the oral bioavailability of CBD in humans is relatively low (<10–20%), largely due to its poor aqueous solubility and significant first-pass hepatic metabolism [38,39], resulting in inconsistent and often subtherapeutic plasma levels. Together, these pharmacokinetic constraints emphasize the importance of conducting in vitro studies under physiologically relevant, serum-containing conditions to avoid overestimating therapeutic potential.

Once absorbed, CBD’s highly lipophilic and redox-active chemical structure (Supplementary Figure S1) enables efficient cellular uptake and direct modulation of intracellular processes critical for cell survival and proliferation [4,16], beyond classical receptor-mediated mechanisms. From a mechanistic perspective, our data point to modulation of intracellular redox homeostasis as a central component of CBD action in RCC cells. CBD altered intracellular ROS/RNS levels in a time- and concentration-dependent manner. At early time points, CBD reduced ROS/RNS levels, consistent with its recognized antioxidant activity, which may involve direct scavenging of reactive species and/or transient activation of cellular antioxidant defenses [1,40,41]. In contrast, prolonged exposure resulted in a progressive increase in ROS/RNS, indicating a shift toward a pro-oxidant state. These temporal dynamics suggest an initial adaptive antioxidant response, followed by redox imbalance as metabolic and mitochondrial stress accumulate. Although sustained ROS/RNS accumulation following CBD treatment is consistent with pro-oxidant mechanisms reported in other cancer models [4], the present study does not establish a direct causal relationship between ROS induction and cell death in RCC cells. Consequently, any involvement of mitochondrial dysfunction, apoptotic signaling, or related stress pathways should be regarded as inferential and hypothesis-generating rather than mechanistically demonstrated. Nevertheless, this later pro-oxidant shift is biologically relevant, as excessive ROS/RNS production is known to disrupt mitochondrial membrane potential and activate caspase-dependent apoptotic pathways. These observations are compatible with prior reports showing that CBD triggers mitochondrial dysfunction, ER stress, and activation of PI3K/Akt/mTOR and MAPK/ERK signaling pathways, ultimately leading to apoptosis and autophagy [3]. In agreement with this proposed mechanism, CBD-induced ROS accumulation has been shown to be required for apoptosis in breast cancer cells, as pharmacological ROS blockade inhibited both apoptotic and autophagic responses [42]. Across multiple cancer models, including breast cancer, leukemia, and glioblastoma, CBD has been reported to disrupt mitochondrial membrane potential, elevate cytosolic and mitochondrial Ca^2+^ levels, and promote early ROS generation, events that collectively activate intrinsic apoptotic signaling pathways [8,16,43,44,45]. These previously described mechanisms are consistent with the redox imbalance and metabolic stress responses observed in RCC cells in the present study, supporting a broader role for CBD as a context-dependent modulator of cancer cell redox biology.

The relevance of this redox-perturbing mechanism is further underscored by the distinctive metabolic features of RCC. RCC exhibits profound metabolic reprogramming, including glutamine addiction, and elevated glutathione-based antioxidant capacity, enabling tumor cells to withstand oxidative stress and resist therapy [46,47,48,49]. By driving ROS/RNS overproduction, CBD may overwhelm this enhanced antioxidant buffering capacity and thereby exploit a metabolic vulnerability intrinsic to RCC, consistent with pro-oxidant and pro-apoptotic mechanisms described in other cancer types [3,42].

A major finding of our study is the comparable sensitivity of malignant RCC cells and non-tumoral HK-2 renal epithelial cells to CBD, indicating limited tumor selectivity under the in vitro conditions employed. However, this apparent lack of selectivity must be interpreted within the constraints of the experimental model used. The present study was conducted in two-dimensional (2D) monocultures, which are suitable for hypothesis-generating investigations but do not recapitulate key features of the in vivo renal microenvironment, including tissue architecture, cell–cell interactions, drug metabolism, and exposure dynamics [50,51]. As a result, cytotoxic responses observed in immortalized renal epithelial cells grown in 2D culture may not reliably predict renal toxicity in vivo. Indeed, despite the relatively high concentrations of CBD required to elicit cytotoxic effects in vitro, clinical studies have consistently reported favorable tolerability profiles, even at high oral doses (up to 1500 mg/day) [36,37,52,53], including in individuals with mild to severe renal impairment [54]. These studies have not identified significant safety concerns, serious adverse events, or clinically relevant laboratory abnormalities. Together, these observations suggest that systemic toxicity, including renal toxicity, may not directly mirror in vitro cytotoxicity profiles, highlighting the importance of cautious interpretation when extrapolating preclinical findings to clinical contexts.

To translate these mechanistic insights into meaningful therapeutic potential for RCC, several limitations must be addressed in future studies. First, CBD’s poor bioavailability and extensive serum binding necessitate the development of improved delivery platforms, such as lipid nanoparticles or nanoemulsions, to enhance systemic exposure and tumor accumulation. Targeted delivery approaches, including ligand-conjugated nanoparticles or renal tumor-specific carriers, may further improve selectivity while minimizing off-target toxicity [55,56,57]. Second, the present study relies primarily on metabolic (MTT) and redox-based assays, which provide useful information but cannot reliably differentiate between growth arrest, reduced metabolic activity, and true cell death. Additional functional validation, including apoptosis markers, cell cycle analysis, and clonogenic survival assays, will be required to strengthen biological interpretation and reduce reliance on metabolic readouts alone. Accordingly, the present findings should be regarded as preliminary and hypothesis-generating, providing a foundation for deeper mechanistic investigation. Third, validation in more physiologically relevant preclinical models is required. Advanced systems such as 3D cultures, organoids, co-culture systems incorporating stromal and immune components and patient-derived xenografts are required to validate these findings and to better assess the translational relevance and therapeutic potential of CBD in RCC. In this context, the impact of hypoxic conditions—an intrinsic feature of RCC driven by VHL/HIF dysregulation—on CBD responsiveness remains an important open question. Finally, the high concentrations required to achieve anticancer effects in vitro, together with the lack of tumor selectivity and attenuation of activity under serum-containing conditions, indicate that CBD is unlikely to be clinically viable as a standalone monotherapy for RCC. Future studies should therefore prioritize strategies aimed at improving tumor specificity, including formulation-based approaches (e.g., nanoparticles or liposomes) and rational combination regimens that permit dose reduction. In particular, CBD may hold promise in combination with established RCC therapies, such as tyrosine kinase inhibitors or immunotherapies, by potentiating oxidative stress and apoptotic signaling, potentially enabling effective antitumor activity at lower, clinically achievable CBD concentrations and representing a promising avenue for future investigation.

## 5. Conclusions

This study provides the first evidence that CBD exhibits anticancer-associated cellular effects in RCC cells by reducing viability and proliferation and by disrupting intracellular redox balance in vitro. However, these effects are highly dependent on serum conditions, require concentrations exceeding those currently achievable in clinical settings, and lack tumor selectivity in simplified cell culture models, thereby limiting immediate translational relevance. Consequently, the mechanistic insights presented here should be regarded as preliminary and primarily intended to guide future research rather than support direct clinical application. Nevertheless, given the established role of metabolic reprogramming and oxidative stress regulation to therapy resistance in RCC, the CBD-induced alterations in metabolic activity and redox homeostasis observed in this study provide a rationale for further investigation using physiologically relevant models, optimized delivery strategies (e.g., nanotechnology-based formulations), and combination approaches aimed at enhancing tumor targeting, bioavailability, and therapeutic selectivity.

## Figures and Tables

**Table 1:** IC_50_ values for CBD-induced cytotoxicity in Caki-1, 769-P, and HK-2 cells under serum-free (0% FBS) and serum-supplemented (5% FBS) conditions after 24 h and 48 h of exposure, as determined by the MTT assay.

	IC_50_ (µM)					
	24 h			48 h		
Cell Line	0% FBS	5% FBS	*p*	0% FBS	5% FBS	*p*
Caki-1	6.87 ± 1.09	14.80 ± 1.10	0.0018	6.87 ± 1.07	11.17 ± 1.13	0.0059
769-P	6.84 ± 1.06	20.05 ± 1.06	<0.0001	5.85 ± 1.08	15.03 ± 1.09	<0.0001
HK-2	5.18 ± 1.08	14.53 ± 1.06	<0.0001	4.88 ± 1.07	12.08 ± 1.12	0.0018

Data are presented as mean ± SEM from three independent experiments performed in duplicate. *p* values for global fit comparisons between serum conditions (0% vs. 5% FBS) are shown.

## Data Availability

The original contributions presented in this study are included in the article. Further inquiries can be directed to the corresponding author.

## Supplementary Materials

The following supporting information can be downloaded at: https://www.mdpi.com/article/10.3390/jcm15020792/s1, Figure S1: Chemical structure of cannabidiol (CBD); Figure S2: Nonlinear regression models for the cell death induced by CBD (1–100 µM) in Caki-1, 769-P, and HK-2 cells, under serum-free (0% FBS) and serum-supplemented (5% FBS) conditions, as evaluated by the MTT assay, after 24 h and 48 h of exposure. Dotted lines represent the 95% confidence band of each fit. Results were obtained from three independent experiments, performed in duplicate. Embedded tables display the estimated IC_50_ values for CBD at the respective cell line and exposure condition, as well as *p* value for group comparison (24 vs. 48 h) of global fits.

## Abbreviations

The following abbreviations are used in this manuscript:

CBD	Cannabidiol
H_2_DCFDA	2,7-Dichlorodihydrofluorescein diacetate
DMEM	Dulbecco’s Modified Eagle Medium
DMSO	Dimethyl sulfoxide
FBS	Fetal bovine serum
IC_50_	Concentrations inhibiting 50% of cell viability
MTT	3-(4,5-Dimethylthiazol-2-yl)-2,5-diphenyltetrazolium bromide
PPARs	Peroxisome proliferator-activated receptors
RCC	Renal cell carcinoma
RNS	Reactive nitrogen species
ROS	Reactive oxygen species
SEM	Standard error of the mean
THC	Δ^9^-Tetrahydrocannabinol
TRP	Transient receptor potential
