# Supplementary material for: Anticancer Potential of Cannabidiol in Renal Cell Carcinoma: Serum Modulation and Preliminary Mechanistic Insights
Source: J Clin Med. 2026 Jan 19;15(2):792. doi: 10.3390/jcm15020792 (PMC12842230; doi:10.3390/jcm15020792)
Supplement: Supplementary file 1 [file jcm-15-00792-s001.zip › jcm-4069604-supplementary.pdf]

Supplementary Material

Anticancer Potential of Cannabidiol in Renal Cell Carcinoma: Serum Modulation and Preliminary Mechanistic Insights

Débora Sousa [1], Filipa Amaro [2,3], Ana Margarida Araújo [4,*], Márcia Carvalho [1,4,5,*]

[1] Instituto de Investigação, Inovação e Desenvolvimento Fernando Pessoa (FP-I3ID), Fundação Ensino e Cultura Fernando Pessoa, Fernando Pessoa University, Praça de 9 de Abril 349, 4249-004 Porto, Portugaly, 4200-150, Porto, Portugal; debora.beatriz.sousa@gmail.com (D.S.)

[2] Associate Laboratory i4HB – Institute for Health and Bioeconomy, University of Porto, 4050-313 Porto, Portugal; up201608003@up.pt (F.A.)

[3] UCIBIO – Applied Molecular Biosciences Unit, Laboratory of Toxicology, Department of Biological Sciences, Faculty of Pharmacy, University of Porto, 4050-313 Porto, Portugal

[4] Laboratório Associado para a Química Verde/Rede de Química e Tecnologia (LAQV/REQUIMTE), Laboratory of Bromatology and Hydrology, Department of Chemical Sciences, Faculty of Pharmacy, University of Porto, 4050-313 Porto, Portugal

[5] RISE-Health, Faculty of Health Sciences, Fernando Pessoa University, Fernando Pessoa Teaching and Culture Foundation, Rua Carlos da Maia 296, 4200-150 Porto, Portugal

* Correspondence: amaraujo@ff.up.pt (A.M.A.); mcarv@ufp.edu.pt (M.C.)

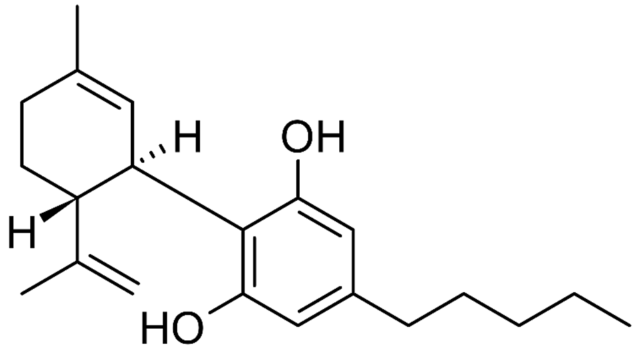

Figure S1. Chemical structure of cannabidiol (CBD)

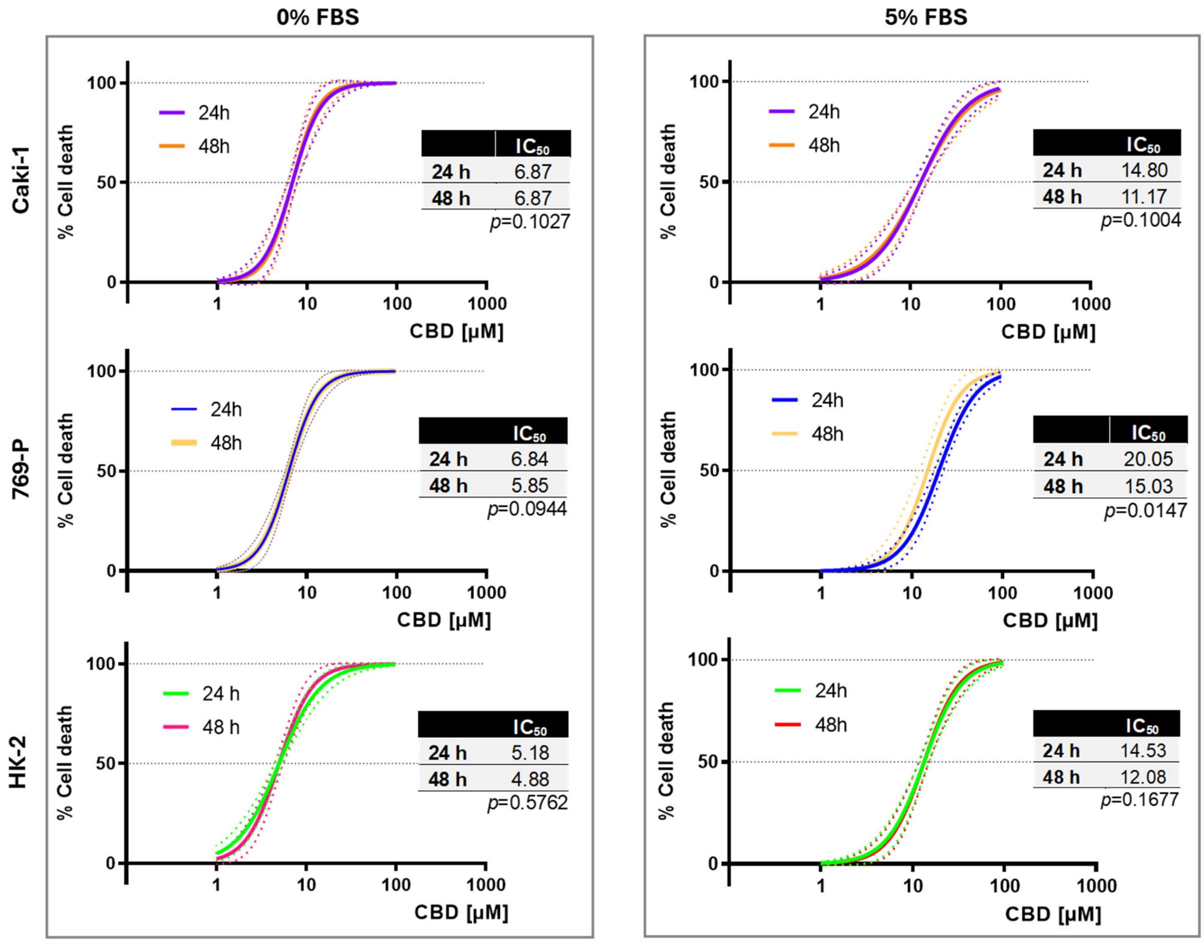

Figure S2. Nonlinear regression models for the cell death induced by CBD (1–100 μM) in Caki-1, 769-P, and HK-2 cells, under serum-free (0% FBS) and serum-supplemented (5% FBS) conditions, as evaluated by the MTT assay, after 24 h and 48 h of exposure. Dotted lines represent the 95% confidence band of each fit. Results were obtained from three independent experiments, performed in duplicate. Embedded tables display the estimated IC_{50} values for CBD at the respective cell line and exposure condition, as well as p value for group comparison (24 vs. 48 h) of global fits.
